# Permanent night work and risk of injuries: A register-based cohort study using payroll data

**DOI:** 10.5271/sjweh.4291

**Published:** 2026-07-01

**Authors:** Kirsten Nabe-Nielsen, Anders Aagaard, Ann Dyreborg Larsen, Helena Breth Nielsen, Johnni Hansen, Åse Marie Hansen, Henrik Albert Kolstad, Jesper Medom Vestergaard, Anne Helene Garde

**Affiliations:** 1 National Research Centre for the Working Environment, Copenhagen, Denmark.; 2 Department of Public Health, University of Copenhagen, Copenhagen, Denmark.; 3 Danish Cancer Institute, Copenhagen, Denmark.; 4 Department of Occupational and Environmental Medicine, Danish Ramazzini Centre, University of Aarhus, Aarhus, Denmark.; 5 Department of Occupational and Environmental Medicine, Danish Ramazzini Centre, Goedstrup Hospital, Herning, Denmark.

**Keywords:** accident, care worker, health professional, nurse, shift work, rotating shift

## Abstract

**Objective:**

Shift work is associated with a higher injury risk, but the optimal way of organizing night work remains debated. This study examined whether the injury risk among permanent night workers differs from that of employees working other types of work schedules with or without night work.

**Methods:**

This register-based cohort study used payroll data from the Danish Working Hour Database over a 12-year period (2007–2018), with daily information on working hours among all hospital employees in Denmark. Work schedules were categorized according to the proportion of night, evening, and day shifts worked in the preceding 365 days. Hospital-treated injuries were identified using the Danish National Patient Register. Poisson regression with generalized estimating equations was used to estimate incidence rate ratios (IRR) for injuries across work schedules. Main analyses were adjusted for sex, age, and job type.

**Results:**

Among 192 711 employees contributing 298.5 million observation days, we identified 87 185 injuries. Permanent night workers had a lower injury risk compared with all other groups of shift workers and a similar risk as permanent day workers. Relative to permanent night workers, the observed injury risk was higher among evening/night workers [IRR 1.37, 95% confidence interval (CI) 1.23–1.53] and day/evening/night workers (IRR 1.37, 95% CI 1.28–1.47).

**Conclusion:**

Permanent night workers had lower risk of injuries than permanent evening workers and workers in 2- or 3-shift schedules. Differences in tasks, adaptation, and selection may contribute to this pattern. Injury prevention efforts should prioritize workers exposed to night shifts in combination with other shift types.

Night work affects 24% of the workforce in the EU (1) and is associated with adverse health outcomes, including injuries (2–5). The elevated injury risk may be attributed to the effects of night work on sleep and cognitive functioning, as understood within the frameworks of homeostatic sleep pressure and circadian regulation (6). First, night work leads to prolonged wakefulness followed by shorter and less refreshing daytime sleep (7), with sleep deprivation leading to cognitive impairments, eg, decreased attention and poorer executive functioning (8). Second, being awake and active during the biological night means that critical tasks are performed during the circadian nadir of alertness (6). In addition, both physical and mental fatigue related not only to the timing, but also the number of consecutive shifts, shift duration and possibilities for rest breaks seem to play a role for the injury risk (9).

Additionally, other characteristics of night shift schedules may determine the effect of night work on adverse outcomes, particularly on sleep (10). For example, it is debated whether permanent night work yields a lower risk of adverse outcomes due to fewer intermittent shifts in the circadian rhythms (11–13). Consequently, permanent night workers may have better possibilities of adapting their circadian rhythm to night work compared with 2- or 3-shift workers whose schedules alternate between day/evening and night work (14). Indeed, studies find that sleep after night shifts is longer in permanent night shift systems than in rotating shift systems (15–18). Interestingly, permanent night workers also report attitudes toward their work schedule that are almost as positive as those reported by permanent day workers (19). Still, it has been estimated that only 21% of the permanent night workers adjust their circadian rhythm enough to benefit from it (14), and in a prior study, sleep after night shifts did not differ significantly between permanent night workers and 3-shift workers (20).

The dilemma of balancing adaptation to night work versus frequent shifts in diurnal rhythms necessitates an investigation of injury risks among permanent night workers compared with employees with other night shift schedules. While studies have described the elevated injury risk among shift workers or after evening and night shifts compared with day shifts (21–25), only a few studies distinguish between permanent night work and 2- or 3-shift work with night shifts. Reviews of working hours and safety incidents conclude that rotating night shift work and non-standard working time arrangements including evening and night shifts encompass a higher injury risk than day work (5, 26). Only a few studies report the injury risk specifically among permanent night workers exclusively in comparison with permanent day workers. The findings from these studies were mixed, as one study reported a higher risk among individuals with self-reported “routine” night work (27), whereas other studies reported insignificantly higher injury risks associated with permanent night work (28, 29), and one study found an insignificantly lower risk among participants with night work (30). Thus, it is unknown if the benefit of adaptation to night work in permanent night workers outweigh the possible accumulation of sleep debt.

Permanent night workers represent a relatively small group in the overall labor market, yet they play a crucial role in covering a significant proportion of night shifts, thereby alleviating the burden of other night workers, for whom night shifts may be less desirable. Insights into health effects of permanent night work are essential to determine if this schedule is sustainable in organizations with 24-hour operations, such as the hospital sector. To address this knowledge gap, we used a cohort of hospital employees to investigate whether the injury risk among permanent night workers differed from that of employees with other shift work schedules or permanent day work. Despite evidence for differences in both directions, previous reviews conclude that night shift work entails a higher injury risk, suggesting that potential adaptation effects may be outweighed by the effects of sleep deprivation and cognitive impairment. Therefore, our *a priori* hypothesis was that permanent night workers had a higher injury risk.

## Methods

### Study design and study population

In this study, each employee’s work-schedule group was assessed using objective payroll data on working hours during the preceding 365 days. As working a night shift may imply an acute injury risk and permanent night work implies a higher likelihood of recent night shifts than other categories of shift work, we assessed the injury risk associated with different work schedules with and without accounting for work-schedule characteristics during the preceding seven days. Furthermore, to improve the comparability between exposed and unexposed groups and reduce the risk of residual confounding, we conducted separate analyses of nurses and assistant nurses, which are the two largest job groups in the hospital sector and those who most frequently have a permanent night work schedule.

Data were compiled from the Danish Working Hour Database (DAD), which holds day-to-day information on exact working hours from all 343 620 individuals employed in one of the five Danish hospital regions in 2007–2020 (31). The regions are administrative entities responsible for the public hospitals. Data include nurses, assistant nurses, physicians, administrative workers, and others. For each individual, DAD holds the exact information of every shift start and shift end date and time, along with background information on sex, age, region, and job type. Using the Danish personal identification number, applied to all residents since 1968, work schedules were linked at the individual level to information on injuries from the National Danish Patient Register (32) for which we had data until 2018.

### Inclusion and exclusion criteria

**Figure 1:**
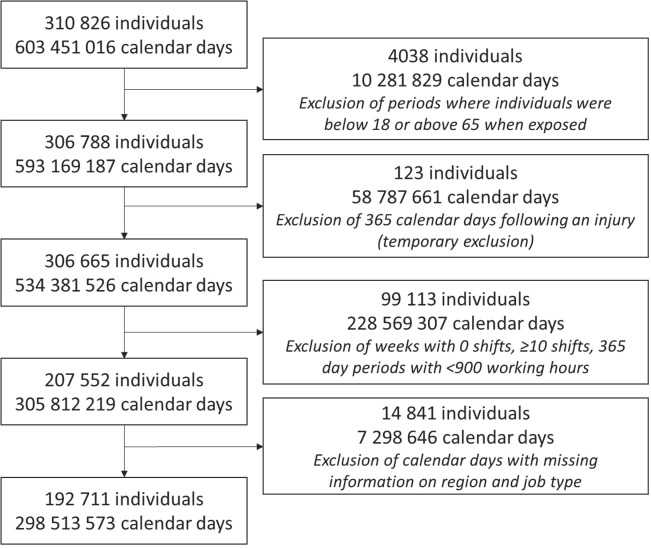
Flow diagram of the inclusion and exclusion of observations from the Danish Working Hour Database.

Based on DAD data for 2007–2018, we initially identified 310 826 unique individuals contributing 603 451 016 observed calendar days. We applied the following exclusion criteria: (i) periods where individuals were <18 or >65 years, (ii) continuous 365-day periods following an injury, (iii) weeks with 0 or ≥10 shifts, (iv)continuous 365-day periods with <900 working hours, and (v) periods with missing data on region or job type. The final sample comprised 192 711 unique individuals contributing 298 513 573 observed calendar days (figure 1). This corresponds to a mean follow-up time for each individual of 4.2 years. The distribution was skewed, though, with a median of 3.7 years (lower 25% percentile=1.3; upper 75% percentile=7.0). This follow-up time could consist of several shorter periods, for example in the case of a temporary 365-day exclusion due to an injury. Individuals were lost to follow-up if they ceased working in one of the Danish regions.

### Assessment of shift types, work-schedule groups, and recent shift work

For each individual and calendar day, we looked 365 days back in time to summarize the types of shifts the person had been working. We did this in order to create work-schedule groups, ie, the main exposure variable. Individuals could change group over time.

We defined a day shift (D) as ≥ hours of work between >06:00 and <21:00, an evening shift (E) as ≥hours of work between ≥18:00 and <02:00, and a night shift (N) as ≥ hours of work between ≥23:00 and ≤06:00. These are the same definitions as used in a related study with the same data (31). A shift could fall within more than one shift definition, eg, 24-hour shifts. In such cases, night shifts were prioritized highest and day shifts were prioritized the lowest, ie, a 24-hour shift would be categorized as a night shift.

**Figure 2:**
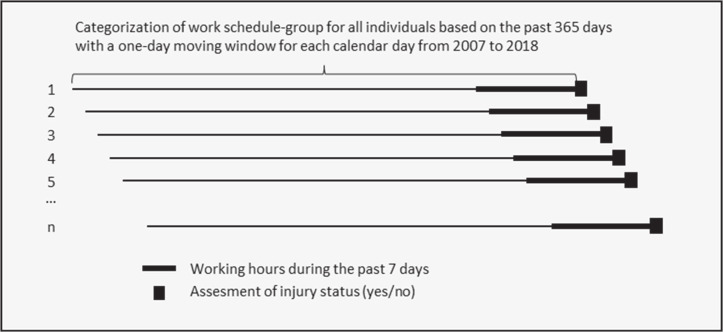
Illustration of study design. For each calendar day, the work schedule during the preceding 365 days was categorized based on summary measures of the distribution of day, evening and night shifts. Furthermore, the work-schedule characteristics during the preceding week are summarized for the additional analyses. The injury status on next calendar day is used as outcome.

Using a one-day running window, employees were categorized into work-schedule groups using information about the shifts worked within the past 365 days in 2007–2018. Hence, for all included individuals, we repeatedly assessed their work schedule during the preceding 365 days. To allow for day-to-day changes in work-schedule group, a running window moving one day at a time was created (figure 2). We applied the following work-schedule groups: *Permanent day*: <6.7% E and <6.7% N*; permanent evening*: <6.7% D and <6.7% N; *permanent night*: <6.7% D and <6.7% E; *day/evening*: ≥6.7% D, ≥6.7% E, and <6.7% N; *day/night*: ≥6.7% D, <6.7% E, and ≥6.7% N; *evening/night*: <6.7% D, ≥6.7% E, and ≥6.7% N and *day/evening/night*: ≥6.7% D, ≥6.7% E and ≥6.7% N (31). For an employee with 150 shifts per year, this cut point (<6.7%) corresponds to working <10 of a specific shift type during that year (31).

### Injuries

Information about injuries was obtained from the Danish National Patient Register (32), which consists of all contacts with the secondary health care system. We did not differentiate between injuries occurring at work and at home, as a sleepiness-related injury risk could carry over from work to home (or vice versa). Furthermore, although some injury types could be more frequent in relation to some shifts compared with others, we wanted to estimate the overall injury risk. Injuries were defined as ICD-10 codes: S0-S99 (injury, poisoning and certain other consequences of external causes), T0-79 (various subcategories of injuries and trauma), T90-98 (sequelae of injuries, of poisoning and of other consequences of external causes), and Y0-09 (various types of assaults) or if the main reason for contact was *accident* necessitating treatment for an acquired injury. Each individual could contribute with more than one injury to the analyses outside the 365-day temporary exclusion period after an injury. We registered 87 185 injuries eligible for inclusion in the analyses.

### Covariates

Not accounting for sex, age and socioeconomic status (eg, in terms of occupational group) can introduce confounding in studies of working time arrangements and injuries (5). Thus, as exposure to night work differs between sexes, across age groups, and job types, we included covariates based on data from DAD: (i) sex (M/F); (ii) age in years (continuous); and (iii) job type (based on DISCO-codes; nurses, assistant nurses, porter, midwives, medical doctor, and other – the latter category contained mainly administrative and technical staff). To account for potential acute effects of shift work characteristics on the injury risk, we included the number of day, evening, and night shifts worked during the seven days preceding the day of a potential injury (continuous). Additionally, to consider that other aspects of the work schedule might also acutely influence injury risk, we included the frequency of long shifts (≥9 hours, dichotomous), frequency of quick returns (<11 hours between two shifts, dichotomous), and average shift length (as continuous variables) over the same period.

### Statistical methods

First, we described the distribution of sex, age, job type, and work-schedule characteristics across observation days. Second, we modelled the association between work-schedule groups based on the shifts worked 365 days back in time and injury status on the calendar day following the end of each 365 day-periods. Following this procedure, we ensured temporal separation of exposure and outcome assessment. Because the study aimed to evaluate whether injury risk differed between permanent night workers and employees with other work schedules, permanent night workers were specified as the reference group in the regression analyses.

Results were presented from three models: model 1 was unadjusted; model 2 (main model) was adjusted for sex, age, and job type; and model 3 was adjusted for the variables included in model 2 and last week’s average shift length, quick returns, long shifts, and number of day, evening and night shifts. In additional analyses, we further explored the injury risk in the two job groups with a sufficiently large number of permanent night workers to allow for meaningful stratified analyses, namely nurses and assistant nurses with adjustment for the same variables as in model 2.

We used Poisson regression with generalized estimating equations (GEE) for all analyses. With this approach, we estimated population-averaged effects, reflecting the study’s aim to inform injury prevention strategies at the workforce level. Using this statistical model, we account for the fact that each employee contributed multiple observation days and adjusted for the correlation between repeated measures from the same individual. We assumed an exchangeable correlation structure, meaning that all repeated measures from the same person were assumed to be equally correlated. To account for differences in the amount of time each person was observed, we included the logarithm of person-days as an offset in the model. This allowed us to estimate injury rates per person-day. We present the incidence rate ratios (IRR) with their 95% confidence interval (CI) for both the main and additional analyses. Data management and descriptive statistics were done in R 4.4.1 and all other statistical analyses were conducted in SAS 9.4.

## Results

In this register-based study, 192 711 individuals contributed 298.5 million observation days (tables 1 and 2). Permanent evening workers were more frequently women and had a higher mean age than individuals in other work-schedule groups. The highest proportion of men was found among permanent night workers and day/night-workers. The majority of participants were nurses or assistant nurses. However, these occupations were underrepresented among permanent day workers. Employees working day/night shifts more often had long shifts, yielding a higher mean shift length. Those working day/evening shifts had slightly more quick returns compared with the other groups. During the seven days preceding the day of potential injury, permanent night workers worked an average of 3.4 night shifts, whereas the other groups worked an average of 1.0 (day/night workers), 1.8 (evening/night workers), or 0.7 (day/evening/night workers) night shifts.

**Table 1:** Description of the characteristics of the data expressed as the number of person days across categories of exposure groups and covariates .

		Total		Permanent night		Permanent day		Permanent evening		Day/evening		Day/night		Evening/night		Day/evening/ night
		N (%)		N (%)		N (%)		N (%)		N (%)		N (%)		N (%)		N (%)
Observations (days)		298 513 573		3 741 683		169 339 549		3 963 456		61 800 85		23 035 343		1 374 686		35 257 997
Observation (years)		817 845 (100)		10 251 (1.25)		463 944 (56.73)		10 859 (1.33)		169 317 (20.70)		63 111 (7.72)		3 766 (0.46)		96 597 (11.81)
Sex																
	Women	237 359 976 (79.51)		2 760 588 (73.78)		132 327 901 (78.14)		3 494 309 (88.16)		51 115 819 (82.71)		16 716 052 (72.57)		1 061 217 (77.2)		29 884 090 (84.76)
	Men	61 153 597 (20.49)		981 095 (26.22)		37 011 648 (21.86)		469 147 (11.84)		10 685 040 (17.29)		6 319 291 (27.43)		313 469 (22.8)		5 373 907 (15.24)
Job type																
	Nurses and assistant nurses ^a^	135 534 625 (45.4)		3 316 708 (88.64)		47 652 974 (28.14)		3 552 318 (89.63)		40 855 677 (66.11)		11 560 738 (50.19)		1 119 279 (81.42)		27 476 931 (77.93)
	Other job groups with patient contact	50 586 746 (16.95)		128 694 (3.44)		27 052 216 (15.98)		175 310 (4.42)		7 698 907 (12.46)		10 710 595 (46.5)		144 036 (10.48)		4 676 988 (13.27)
	Administration, service and others	112 392 202 (37.65)		296 281 (7.92)		94 634 359 (55.88)		235 828 (5.95)		13 246 275 (21.43)		764 010 (3.32)		111 371 (8.10)		3 104 078 (8.8)
	Long shift last week (yes)	58 523 067 (19.60)		794 722 (21.24)		19 200 441 (11.34)		558 566 (14.09)		17 452 138 (28.24)		9 168 737 (39.80)		354 749 (25.81)		10 993 714 (31.18)
	Any quick return last week (yes)	28 954 850 (9.7)		196 461 (5.25)		4 870 165 (2.88)		127 007 (3.2)		13 553 059 (21.93)		2 671 602 (11.60)		157 764 (11.48)		7 378 792 (20.93)

^a^ Medical doctors, midwives, porter.

**Table 2:** Description of the characteristics of the data across categories of exposure groups. [SD=standard deviation.]

	Total		Permanent night		Permanent day		Permanent evening		Day/evening		Day/night		Evening/night		Day/evening/ night
	Mean (SD)		Mean (SD)		Mean (SD)		Mean (SD)		Mean (SD)		Mean (SD)		Mean (SD)		Mean (SD)
Age (years)	45.6 (10.4)		49.7 (9.1)		46.9 (9.9)		53.0 (8.4)		45.8 (10.5)		41.4 (9.8)		49.1 (9.4)		40.5 (10.7)
Average shift length last week (hours)	7.8 (1.6)		8.3 (1.3)		7.4 (1.0)		8.1 (0.7)		8.0 (1.2)		9.1 (4.0)		8.4 (1.3)		8.4 (1.6)
Average number day shifts last week	3.3 (1.7)		0.1 (0.3)		4.2 (1.2)		0.1 (0.3)		2.5 (1.6)		2.5 (1.6)		0.1 (0.4)		2.0 (1.5)
Average number evening shifts last week	0.4 (0.9)		0.0 (0.2)		0.0 (0.2)		3.2 (1.4)		1.1 (1.2)		0.1 (0.3)		1.6 (1.6)		0.8 (1.1)
Average number night shifts last week	0.2 (0.8)		3.4 (1.6)		0.0 (0.1)		0.0 (0.2)		0.0 (0.3)		1.0 (1.2)		1.8 (1.8)		0.7 (1.1)

The results of the analyses examining the association between work schedule and injury risk are shown in table 3. Using *permanent night workers* as the reference group, the crude model showed a significantly lower injury risk among permanent day workers and significantly higher injury risk among all other groups of shift workers (model 1). In model 2, compared with permanent night workers, all other groups of shift workers showed a higher injury risk. The highest IRR were observed among evening/night workers (IRR 1.37, 95% CI 1.23–1.53) and day/evening/night workers (IRR 1.37, 95% CI 1.28–1.47). In model 3, individuals with permanent schedules (day, evening, or night) exhibited similar injury risks, whereas the estimates for shift workers with combinations of shift types were attenuated but remained significantly higher than the estimate for permanent night workers.

**Table 3:** The association between work schedule during the preceding 365 days and risk of injury. [IR=incidence rate; IRR=IR ratio; PY=person years; CI=confidence intervals].

Work schedule-groups	Cases	Person-days	IR per 1000 PY	Model 1 Crude model			Model 2 Main model ^a^			Model 3 Supplementary adjustment ^b^	
				IRR	95% CI		IRR	95% CI		IRR	95% CI
Permanent night	1076	3 741 683	105	1.00			1.00			1.00	
Permanent day	42 921	169 339 549	93	0.88	0.83–0.95		0.99	0.93–1.07		0.93	0.86–1.01
Permanent evening	1251	3 963 456	115	1.10	1.01–1.21		1.17	1.07–1.29		1.02	0.93–1.13
Day/evening	20 384	61 800 859	120	1.16	1.08–1.24		1.22	1.14–1.31		1.09	1.01–1.18
Day/night	7252	23 035 343	115	1.10	1.02–1.18		1.19	1.10–1.28		1.10	1.02–1.19
Evening/night	545	1 374 686	145	1.38	1.23–1.54		1.37	1.23–1.53		1.27	1.14–1.43
Day/evening/night	13 756	35 257 997	142	1.37	1.28–1.47		1.37	1.28–1.47		1.25	1.16–1.35

ª Adjusted for age, sex and job type. ^b^ Adjusted for last 7 days’ average shift length, quick returns, long shifts and number of day, evening and night shifts, age, sex and job type.

In additional analyses restricted to nurses only, we found a moderately higher injury risk among day/evening/night-workers (IRR 1.20, 95% CI 1.06–1.35) compared with permanent night workers. In the sample restricted to assistant nurses, we observed a higher injury risk among all other work-schedule groups with IRR in the range of 1.13–1.51 compared with permanent night workers (table 4).

**Table 4:** The association between work schedule during the preceding 365 days and risk of injury in nurses and assistant nurses, respectively. [IRR=incidence rate ratio; CI=Confidence Intervals].

Work-schedule groups	Nurses ^a^						Assistant nurses ^a^				
	Cases	Person-days	IR per 1000 PY	IRR	95% CI		Cases	Person-days	IR per 1000 PY	IRR	95% CI
Permanent night	331	1 135 545	106	1.00			625	2 181 163	105	1.00	
Permanent day	10 375	39 941 222	95	0.89	0.79–1.00		2556	7 711 752	121	1.15	1.04–1.27
Permanent evening	457	1 736 251	96	0.96	0.82–1.13		621	1 816 067	125	1.31	1.15–1.48
Day/evening	9245	28 113 625	120	1.10	0.97–1.24		4619	1 2742 052	132	1.28	1.17–1.41
Day/night	2704	9 010 307	110	0.96	0.85–1.09		840	2 550 431	120	1.13	1.01–1.27
Evening/night	179	540 358	121	1.14	0.94–1.38		246	578 921	155	1.51	1.29–1.78
Day/evening/night	8776	22 887 931	140	1.20	1.06–1.35		1918	4 589 000	153	1.42	1.28–1.57

^a^ Adjusted for age and sex.

## Discussion

### Main findings

Overall, the injury risk among permanent night workers was comparable to that of permanent day workers. In contrast, all other groups of shift workers exhibited a significantly higher injury risk relative to permanent night workers, and these estimates were not substantially affected by work-schedule characteristics during the preceding seven days. Notably, work schedules that did not involve rotation between shift types appeared to be associated with the lowest injury risk. The highest IRR was observed among individuals working night shifts in combination with evening shifts compared with permanent night workers. In our subgroup analyses, this pattern was not evident among nurses, as injury risks were largely similar across work-schedule groups, with the exception of day/evening/night workers. In contrast, among assistant nurses, all other work-schedule groups demonstrated a higher injury risk compared with permanent night workers.

### Comparison with previous research

Our findings are in accordance with the results of systematic reviews, as we found a higher injury risk among 2- or 3-shift workers than among day workers (5, 26, 33). Importantly, however, this comparison was not the primary focus of the current study and was not formally tested. In alignment with the objective of the present study, we used permanent night workers as the reference group. In our study, permanent day workers and permanent night workers did not differ in injury risk in the adjusted analyses. In that sense, our results differ from previous studies suggesting a higher injury risk among permanent night workers compared with permanent day workers within hospitals [risk ratio (RR) 1.67, 95% CI 0.93–2.93 (28)] and across different industries [RR 1.30, 95% CI 1.12–1.52 (27); RR 1.38, 95% CI 0.95–2.00) (29)] or finding an insignificantly moderately lower risk among healthcare workers with night work [RR 0.81, 95% CI 0.50–1.33 (30)]. Only in the unadjusted analyses, we replicated the previously published elevated injury risk among permanent night workers compared with day workers (27). The main differences between our study and previous research lie in the use of register-based exposure and outcome data, a substantially larger sample size (192 000 versus 730–15 000) encompassing considerably greater statistical power and the ability to separate the effects of long-term work-schedule groups from work-schedule characteristics during the preceding week. Moreover, it is unclear whether earlier studies applied a temporary exclusion period following injuries. Taken together, these distinctions underscore the substantially higher methodological rigor of our study relative to prior work.

Also, consecutive evening or night shifts (24) and quick returns (34) are associated with a higher injury risk in this cohort. However, isolating the effect of specific work-schedule characteristics on health outcomes is a difficult task, as these characteristics are highly intertwined. In the current study, we aimed to elucidate the injury risk among permanent night workers compared with other work-schedule groups thereby capturing the clusters of work-schedule characteristics of these groups. Both with and without adjustment for working hours during the preceding seven days, our overall finding was that permanent night workers experienced relatively fewer injuries resulting in hospital contact compared with colleagues working combinations of shifts. A comparison of the models with and without adjustment for work-schedule characteristics during the preceding seven days did not change this finding, although estimates were attenuated after full adjustment. Our findings support the argument proposed by some scholars that permanent night work may be associated with a lower risk of adverse outcomes, in this case, injuries, compared with other night shift schedules. However, based on our data, we cannot empirically determine whether this association reflects adaptation to night work (eg, improved daytime sleep and greater alertness during and after shifts) or whether it is explained by contextual confounding factors or selection effects.

In a previous study involving participants from multiple industries, no specific type of injury or illness stood out among those with non-standard work schedules compared with permanent day workers. The primary cause in both groups were musculoskeletal conditions, cuts and bruises, and other traumatic injuries (27), and a similar overall pattern was found in health care workers (30). The present study covers all types of injuries, yet, national statistics elucidates that the most work-related injuries with a known cause in the hospital sector are related to contact with electricity, extreme temperatures, or hazardous substances (38%), physical or psychological strain (22%), and falls (12%) (35). While some of these causes may generically apply to the majority of hospital employees, others may differ between job groups due to different job tasks. To indirectly address these contextual factors, we performed the analyses in two large specific job groups, nurses and nursing assistants, with the highest occurrence of permanent night work and with different formal competencies and therefore also somewhat different job tasks. These results showed that the injury risk among nurses is somewhat similar across different work schedules. Only nurses working all three shift types stood out as having a higher injury risk. Yet, the injury risk among assistant nurses is lower in permanent night workers than among all other work-schedule groups. Thus, there appear to be some underlying explanatory factors that causes a differential injury risk across work schedules in different job groups. The reasons underlying these findings remain speculative. However, differences in the distribution of job demands and risky tasks may represent plausible explanations.

### Strengths and limitations

The strengths of the current study are the large study population with no drop-out as long as the individual was employed in a Danish region, and almost 300 million days of observation, the long follow-up and the objective register-based measures of both exposure (working hours) and outcome (injuries). The register-based working hours are used for the calculation of individual salary (ie, payroll data), and both employer and employee have an incentive for correct registration of these (31). The use of a moving window also allows individuals to change exposure group if they alter their work schedule during follow-up. Thus, the risk of misclassification of exposure is reduced, compared with previous studies using a survey-based single time-point assessment of work schedule (5, 26). Furthermore, if an individual changed work schedule over time, the same individual would contribute to more than one exposure group.

Information about injuries originates from hospital registries, and we expect that the injury registration has a low rate of false positive entries. However, particularly minor injuries, which can be handled by the hospital employees themselves, will presumably not lead to contact with the emergency department, and will thus not be registered. This means that this study only elucidates differences in the risk of major injuries, whereas we cannot detect differences in minor injuries and whether this risk differs between shifts. In line with the “common cause” hypothesis (suggesting that minor and major injuries share the same underlying causes), we propose that the observed findings for major injuries requiring hospital contact reflect a broader underlying distribution of near misses, minor injuries, and major injuries across the different work-schedule groups (36). We can only speculate if differential misclassification of the outcome occurs, ie, that, for example, permanent night workers less frequently seek medical care in the case of a less severe injury. This could be the case if their working hours and related contextual factors serve as a barrier to hospital contact. If this is the case, the injury risk in permanent night workers would be underestimated. It was outside of the scope of the present study to analyze different types of injuries, eg, occupational, transport and leisure-time injuries. However, in a previous study in the same population looking at different types of injuries, evening shifts were more commonly followed by occupational injuries, night shifts were less commonly followed by transportation injuries, and evening shifts were less commonly followed by leisure-time injuries compared with day shifts (23).

It is a limitation of the study that we cannot analyze more thoroughly what tasks permanent night workers are responsible for during night shifts compared with other types of employees. Furthermore, other work-related or individual differences could explain differences in injury risk between groups. Such information would be useful in understanding the observed differences, and it would also be instrumental in injury prevention. Our data suggest, however, that the distribution of job tasks and other unmeasured factors with safety implications differs not only between shifts, but also between job groups.

Selection into and out of specific work schedules such as permanent night work, may also influence the findings (37). We expect that a “healthy shift worker effect” may be at play, resulting in a population of permanent night workers who are better able to adapt to frequent night shifts. This selection mechanism may be linked to individual adaptability to night work and could be speculated to contribute to a higher tolerance to shift work and thus a lower injury risk—for example, through higher sleep flexibility, a later chronotype, or the ability to sleep during the day (38, 39). However, factors such as economic incentives, family circumstances, personal resources, or limited job opportunities may also shape work schedule-preferences (20, 40). These factors may, in some cases, “push” individuals into permanent night work despite experiencing negative effects, for example, on sleep, potentially increasing their injury risk.

The hypothesized differences across shifts, jobs and wards in terms of the occurrence of “risky tasks” as well as selection mechanisms into and out of permanent night work and other work schedules could have important consequences for the generalizability and implications of the findings of this study. This means that despite that permanent night workers cannot be identified as a high-risk group in terms of injuries, our findings do not necessarily imply that covering all night shifts by permanent night workers would prevent injuries. This is due to potential selection into permanent night and because of unaccounted differences in factors implying a risk of injury. Furthermore, permanent night workers may be at higher risk of other adverse health outcomes due to accumulation of night work, eg, breast cancer (4), which needs adequate attention and preventive actions. Likewise, permanent night work may entail other consequences, such as reduced opportunities for social participation.

### Concluding remarks

Against our initial hypothesis, we found that permanent night workers displayed a lower injury risk than permanent evening workers and workers in 2- or 3-shift schedules. Particularly, having work schedules encompassing both evening and night shifts appears to be associated a higher rate of injuries requiring hospital contact.

Further studies are needed to disentangle the effects of work tasks or selection, which may have contributed to these findings. More detailed registration of work tasks, events and actions preceding the injuries as well as mixed methods research projects including participant observations could help move the field forward. For now, injury prevention efforts should preferably be targeted at shift workers working combinations of shifts as these groups seem to have a higher injury risk than permanent night workers. More knowledge about root causes is, however, necessary in this process and it should be considered that redistribution of tasks and responsibilities could alter injury patterns among hospital employees.
